# A Chip Antenna for Bluetooth Earphones with Cross-Head Interference Tested from Received-Signal Sensing

**DOI:** 10.3390/s22113969

**Published:** 2022-05-24

**Authors:** Yejune Seo, Junghyun Cho, Yejin Lee, Jiyeon Jang, Hyung-Wook Kwon, Sungtek Kahng

**Affiliations:** 1 Department of Information & Telecommunication Engineering, Incheon National University, Incheon 22012, Korea; m.june@inu.ac.kr (Y.S.); elsa@inu.ac.kr (J.C.); allen@inu.ac.kr (Y.L.); yeon.jj@inu.ac.kr (J.J.); 2 Convergence Research Center for Insect Vectors, Incheon National University, Incheon 22012, Korea; hwkwon@inu.ac.kr

**Keywords:** earphone, wearable antenna, chip antenna, equivalent circuit, metamaterial structure, received signal strength sensing, head-ear-phantom

## Abstract

In this paper, a novel chip antenna and its function in wireless connectivity are presented for Bluetooth (BLT) earphones. The chip antenna is a metamaterial so compact (<λ/8), as the size of 4.9 × 13.0 × 2.0 mm^3^, that when it is mounted on the realistic PCB, it can be held in the enclosure of the BLT earphone. This setting does not degrade the resonance (S_11_ < −10 dB) of the proposed antenna. As two earphones in a pair are demanded to communicate with each other, one shares an RF signal with the other and they take turns as the master and slave. The received signal sensing is conducted with the latest model of human head-ear-phantom located between the earphones to mimic the real use-case and cross-head interference. Electromagnetic simulation of the antenna is done and verified by fabrication and measurement. Particularly, received-signal strength indications between the proposed antennas in the earphones are experimentally obtained as −67.5 dBm and −70 dBm without and with the head-ear-phantom, respectively, much greater than −120 dBm, the limit of detection, and implying acceptable connectivity and invulnerability over cross-head-interference problems.

## 1. Introduction

People wear smartwatches and can check their heartbeats while walking or running. Wearing earbuds, they listen to music wirelessly streamed from their phones. Home appliances are being advertised to do eye-capturing jobs under the name of smart fridges or smart TVs. They are e-Health monitoring, entertainment and home electronics, having smart as the prefix. These subjects are handled in the area of the Internet of Things (IoT) [1].

IoT mixes the strengths of electronics and networking. Sensors and communication modules are distributed in a network and across multiple networks [2,3,4,5,6,7,8]. Software in the IoT equipment watches and controls the data traffic, resource allocation and choice of a network and conversion to different routes. This dynamic networking is operated wirelessly, which means a contribution from RF technologies as part of the electronics for the IoT. Looking back upon what was required in RFID, antennas for tags and the reader in the service play crucial roles. Especially when tags are laid in an unfriendly environment, such as lossy objects or shadows, simple antenna design techniques and just the use of commercial antennas turn out inadequate for a wireless link of reasonable quality.

As the RFID technology revealed limitations, it has pushed wireless service developers to suggest M2M (Machine-to-Machine), D2D (Device-to-Device), and IoE (Internet of Everything) to reach the era of IoT (Internet of Things). Though the previous and current mottos of wireless connectivity do not seem to overlap, there are things to solve in common. Time-varying conditions and signal strength between multiple nodes in a network should be considered and handled seriously. Among a number of approaches to satisfactory communication from the TX-node to the RX-node, advanced antennas play an important role in coping with the problem. Therefore, it is worthwhile to look over the antennas used in sensor networking, BLT services and wireless body-area networking.

Almost all the antennas aimed at IoT applications are basically small. The survey of the related literature shows that a group of antennas is commonly made on the PCB without a housing, which is ideal. As opposed to this, the commercial product needs enclosures. While some antennas are in harsh environmental conditions, such as being near human tissue or having an obstacle in the line of sight, others are not. A circular patch monopole was introduced for body-centric communication by Y. Sun et al. [9]. The antenna could be realized on textiles and tested with bending and crumpling. One presented a dual-mode property to a microstrip by having a meander line inside a loop [10]. The ground plane exists under the two radiating lines to prevent unwanted coupling from a human tissue-mimicking cylinder. Z.-G. Liu et al. etched the metal ground of a microstrip ring antenna similar to parallel rings to generate a dual-band resonance [11]. Though this mentioned body-centric communication, the human phantom test was not done. The top of an antenna was separated from the bottom through the air gap, and there are relatively long vias between the two layers for dual-band on- and off-body communication [12]. It is taller than other wearable antennas and was adopted to a button of shirts worn by a human being. Because they gave enough height to the antenna from the ground, the performance of their antenna was not disturbed by the examinee. A slotted circular patch antenna was worn by a person to see the frequency variation for on-body/off-body communication [13]. PIN diodes were loaded to change the state. A. Arif et al. used a slotted metal ground for a fractal-shaped patch on top for WBAN communication. They observed the effects of human tissue on the antenna performance by attaching it on a person [14]. Having on-body communication in mind, X. Li et al. used two wide planes to feed a bow-tie dipole supported by a partially meandered loop [15]. They did test their antenna without the human phantom but with PEG-water-solution. Instead of the liquid material, an electromagnetic model of the human body holds a tri-band antenna made up of a stub dipole and a slot dipole on a large CPW (coplanar-waveguide) ground [16]. Their antenna was made with RT 5880 as the substrate, however, polytetrafluoroethylene (PTFE) was adopted to go with ink-jet printing for the other body-centric communication case [17]. W. Su et al. printed a cluster of mushrooms on the top layer of PTFE and used in mode-tracking of human activity recognition. It does not handle the RF link whose path includes the head.

Contrary to the work mentioned so far, the spot of interest needs to move from the body to the head for special requirements. A. Cihangir et al. gave a patch-coupled monopole and a loop antenna separately to two ends of a long side of a goggle [18]. This does not work on earphones. They placed the wearable device around the specific anthropomorphic mannequin (SAM) and observed the radiated field patterns. Many researchers have addressed body-centric connectivity with phones, goggles, watches, and tags on conductive textiles, but few have handled antennas for head-sets or ear-sets or earphones, supposed that they are relatively thin and small. An ear-set is observed in the title of Z. Zahid et al.’s work, where they took advantage of speaker wires for their antenna [19]. Their antenna necessitates its own PCB for feeding and ground, which has no choice but to exist outside of the ear-set, which is not appealing to realistic prototyping. S. S. Zhekov wrote a comprehensive review of the electromagnetic characteristics of a variety of commercial earphones [20]. Although they did not make an attempt to design antennas proper for the devices, they conducted interesting experiments on the EIRP (effective isotropic radiated power) of each of the products as well as the electric field intensity when a volunteer wore the DUTs himself. The SAM was not used, but the fact that in-the-ear wearable devices were brought is worth attention.

In this paper, a body-centric communication device fitted into the ear is constructed with a reliable SAM to emulate the real use of earbuds. More concretely, a small antenna is devised to be placed in the mock-up earbud and this structure is placed into the ear phantom as part of the head SAM, which is the most advanced model. The antenna is differentiated from the aforementioned antennas in that it does not require the layer of the wide metal ground or external ground plane and takes up a small area (real-estate) within the earphone. Its size amounts to 4.9 × 13.0 × 2.0 mm^3^, made possible by a metamaterial-inspired configuration. This chip antenna emanates radiated fields of appropriate antenna gain in spite of its small volume. This is noticeably present in the steps of the electromagnetic simulation-based design and the measurement of the fabricated prototype. Additionally, the RSSI test, as the demonstration of real wireless linkage between the left and right earphones stuck in-the-ear phantoms, is conducted. It should be noted, however, that the head phantom blocks the two earphones in this experiment of signal exchange between the left ear to the right ear and vice versa, which is the first attempt in the antennas and propagation society. Through the signal strength and RSSI measurement, wireless connectivity is effectively verified with −70 dBm exceeding −120 dBm as the limit of detection, meaning it overcame the blockage by the head phantom. The theoretical and experimental aspects of the proposed sensing scheme are unfolded from the antenna design and its performance in the next section, through its integration into earphone mock-ups and frequency responses to the RSSI test with the head-ear-phantom in the ending section.

## 2. Design of the Compact Antenna and Its Performances

### 2.1. Initial Step to Shape the Structure with the Left-Handed Metamaterial Equivalent Circuit

Obviously different from mobile handsets or smart glasses, an earphone faces more challenging conditions in design. It is surrounded by human tissue and, really, a small space is given to the antenna placement, as shown in the following diagram.

**Figure 1:**
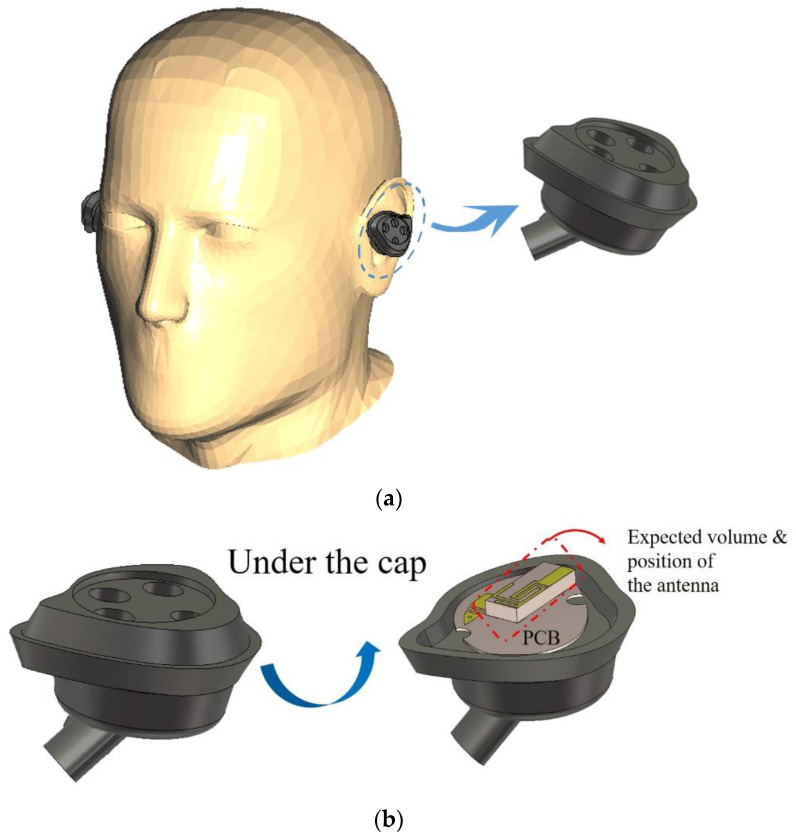
The earphone placed in the ear of the SAM. (**a**) Overall view; (**b**) antenna in the earphone.

Figure 1a shows how the earphone exists on the head phantom. While the human tissue is under the mobile handheld antenna, regarding the present work, there is human tissue around the earphone. This means the antenna for the earphone will be highly likely disturbed by the human tissue, known as a highly lossy material. This drawback should be overcome. The design procedure considers the latest head-ear model, which copies the real human head and ears. For example, there should be an ear meatus or canal and acoustic auricle or tragus/anti-tragus, concha, and helix of a human ear. Accordingly, POPEYE-V10 was procured and available with its computer-aided design model, while other phantoms do not possess ear canals and conchas. In Figure 1b, the antenna yet-to-be-determined is laid inside the earphone. Under the cap of the wearable device, a chip antenna is adequate. At this moment, some might be wondering if the monopole antenna or PIFA may be another candidate for the earphone under discussion [13]. Thus, the geometries of the two kinds of antennas were drawn to check if they met the resonance condition of BLT frequency, and the electromagnetic simulation was conducted as follows below.

**Figure 2:**
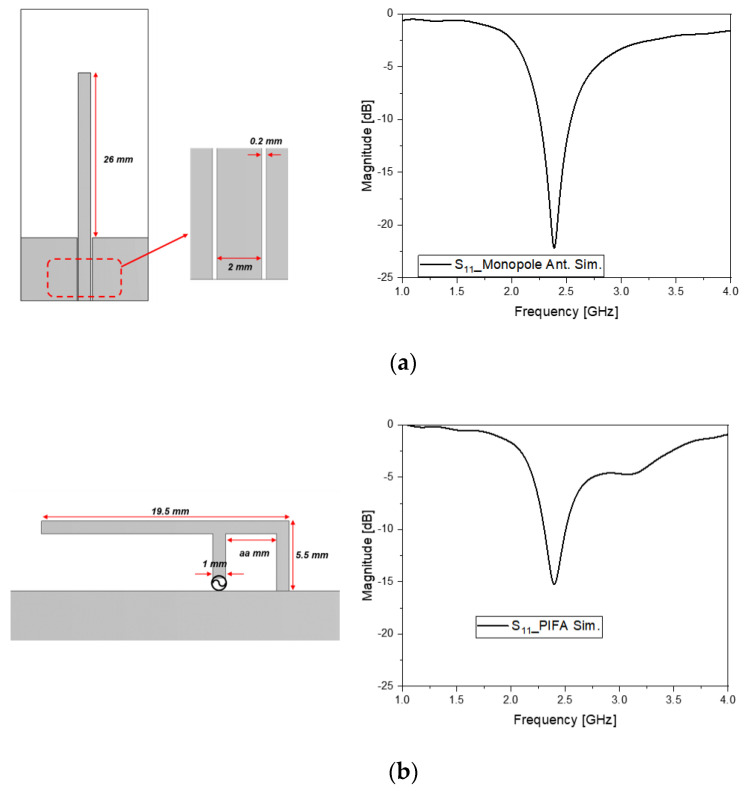
Checking the commonly used antennas. (**a**) Monopole antenna and its S_11_; (**b**) PIFA antenna and its S_11_.

In Figure 2a, a quarter-wavelength stub stems out of the coplanar waveguide feed, easily seen in the PCB and RF connection. When the length of the stub becomes 24 mm, the dip occurs at 2.45 GHz in S_11_. It outgrows the long edge of the PCB under the earphone cap. Besides this shortcoming, the straight line cannot make the monopole stay inside the wearable device. This problem leads to the bending of the straight line as in the PIFA, as shown in Figure 2b. The line of the monopole can be shorter in the PIFA, such as 19.5 mm, for the resonance at 2.45 GHz, as observed in S_11_. This length violates the limit of the area of placement, which is in the proximity of other electronic functional blocks having chipsets, touch-sensors, routing, etc. The drawbacks of the commonly used antennas, given the limited room of the earphone, have driven antenna designers to seek alternative approaches. One approach is the use of metamaterial antennas, in that they are smaller than a half-wavelength with the help of generating zero and negative propagation constants. This left-handed (LH) transmission line (TL) leads to effective size reduction. The LH TL belongs to the composite right- and left-handed (CRLH) circuits [21,22,23]. The following will present the basic CRLH circuit as having an increasing number of parameters and a higher degree of freedom in design that reflects on meaningful size reduction and electromagnetic coupling factors.

**Figure 3:**
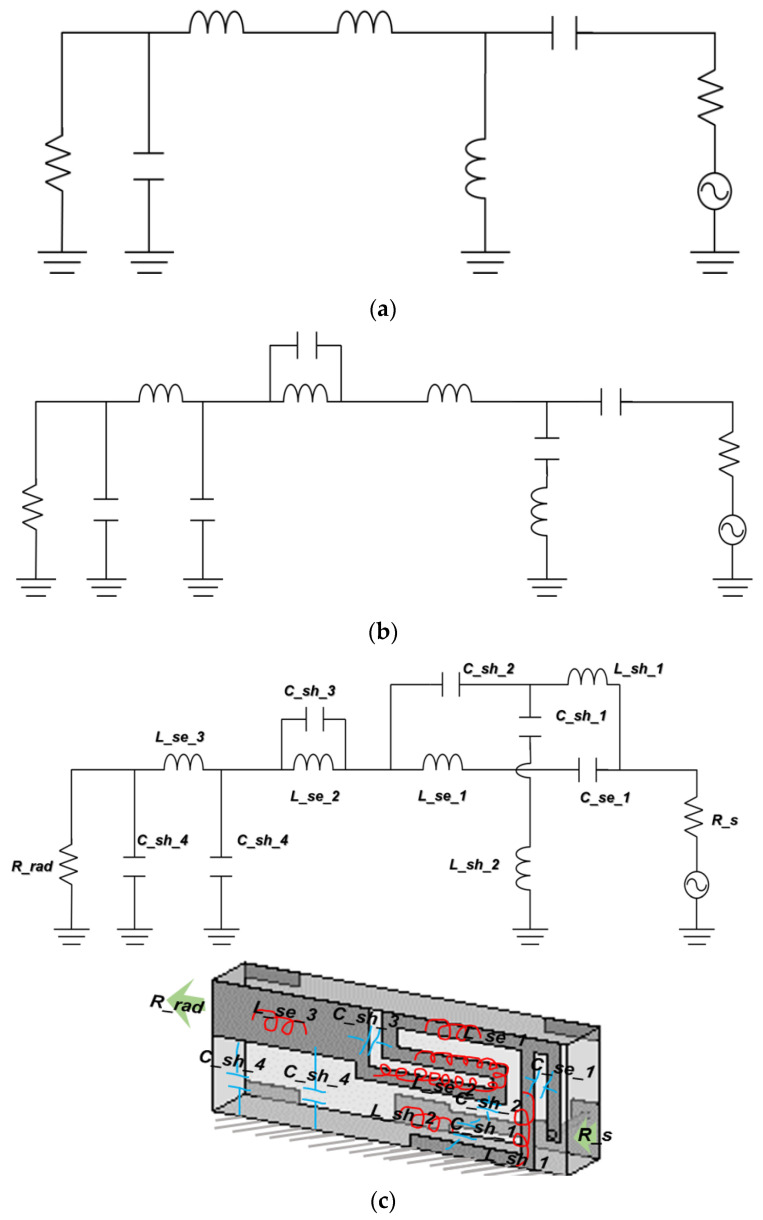

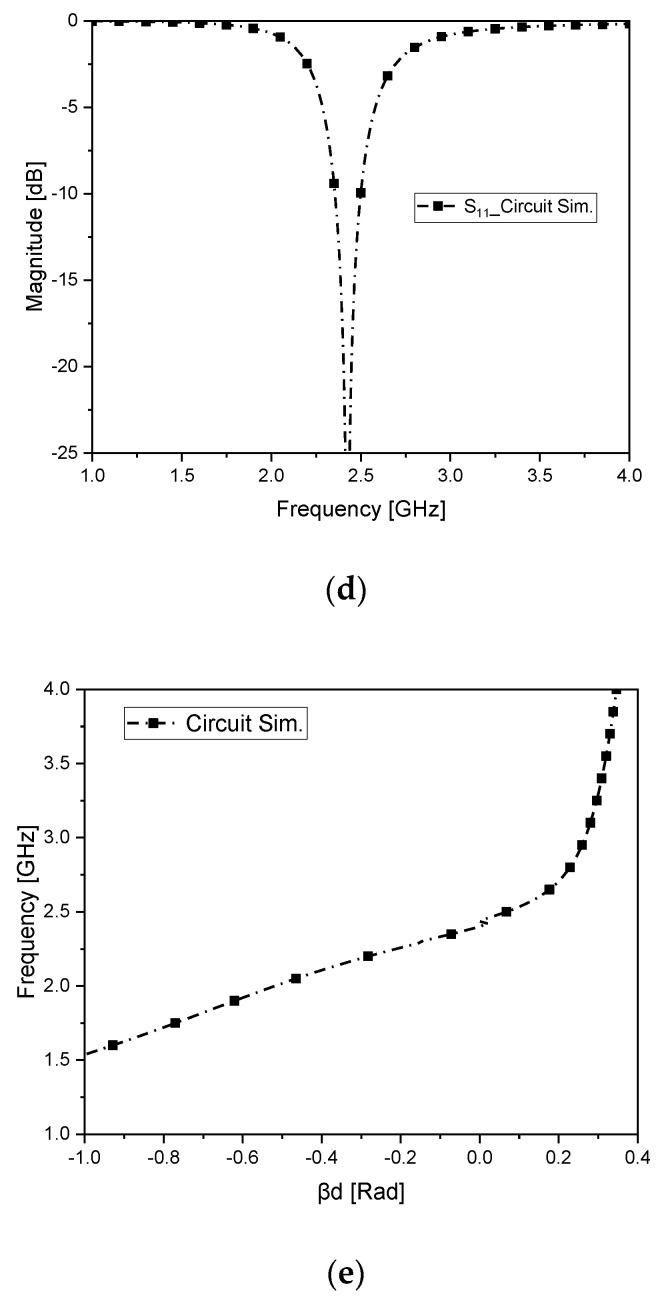
Equivalent circuit setup for a proper antenna. (**a**) Basic CRLH circuit; (**b**) E-CRLH circuit; (**c**) modification of the E-CRLH circuit model for antenna’s size reduction; (**d**) S_11_ of the E-CRLH circuit; (**e**) dispersion diagram of the E-CRLH equivalent circuit.

There are three circuit models in Figure 3. The form of the basic CRLH circuit is made up of a series, such as an inductor (*L*) and capacitor (*C*), and shunt *L* and *C*, as in Figure 3a [22,23,24,25]. With more elements added to the simple CRLH circuit, it can provide more functions, and it is called the extended CRLH (E-CRLH) circuit [26,27]. As shown in Figure 3b, the E-CRLH needs to evolve to one with a higher degree of inter-element coupling generated in a real metal-patterned device. Figure 3c presents an E-CRLH equivalent circuit and the initial shape of its geometrical rendition. The roles of the circuit elements *C_se_1*, *C_sh_2*, *C_sh_4*, *L_se_2*, *L_sh_1*, *C_sh_1*, *C_sh_3*, *L_se_1*, *L_se_3*, *L_sh_2*, *R_s* and *R_rad* can be mapped into inductive or capacitive parts of a dielectric rectangular prism. As is desired for physical miniaturization, the metallic pattern as the current path on the surface of the dielectric prism should be divided into the main line and stubs. The main line of the structure starts from the power supply and *R_s* through *C_se_1*, *L_se_1*, *L_se_2*, *L_se_3*, *C_sh_3* and *C_sh_4* to *R_rad*. *L_sh_2* is with the stub on the top surface. The stub on the bottom surface of the prism consists of *L_sh_2*. The three line segments are coupled through *C_se_1*, *C_sh_1* and *C_sh_2*. More specifically, *C_se_1* occurs between the main path and the upper stub, and *C_sh_1* is the capacitive coupling between the upper and lower stubs. From the lower stub to the main path, the coupling is represented with *C_sh_2*. On the main line, the current flows with inductances *L_se_1*, *L_se_2* and *L_se_3*. The current created on the upper stub goes with *L_sh_2*. The lower stub lets the current flow with *L_sh_1*. These circuit elements are figured out by having their E-CRLH model meet the resonance condition at the target frequency. The values of the inductors and capacitors, at the time, are tabulated as follows.

With the values in Table 1, the equivalent circuit of the antenna is characterized by the input-port reflection coefficient and dispersion diagram. In Figure 3d, the curve of S_11_ as the reflection coefficient has a dip as the resonance at 2.45 GH, which will be shown again by the antenna realized based on this circuit. A proof of metamaterials is the dispersion curve as the propagation constant vs. frequency, and this is plotted in Figure 3e. Zero propagation constant (*β* = 0) is observed at the target frequency, which already tells readers this circuit model is the metamaterial. In addition, the LH region, where *β* < 0, is generated below 2.4 GHz. The physical structure of this circuit is shaped in the following section, where its frequency response and metamaterial properties are continuously investigated.

### 2.2. Shaping the Antenna Structure from the E-CRLH Circuit Model Approach

Ahead of this section, the equivalent circuit was built to resonate at the BLT frequency with the circuit elements in Figure 3c and Table 1. In the phase of realization, the current flowing line segments become the metallic pattern on a rectangular dielectric cube with the circuit elements.

**Figure 4:**
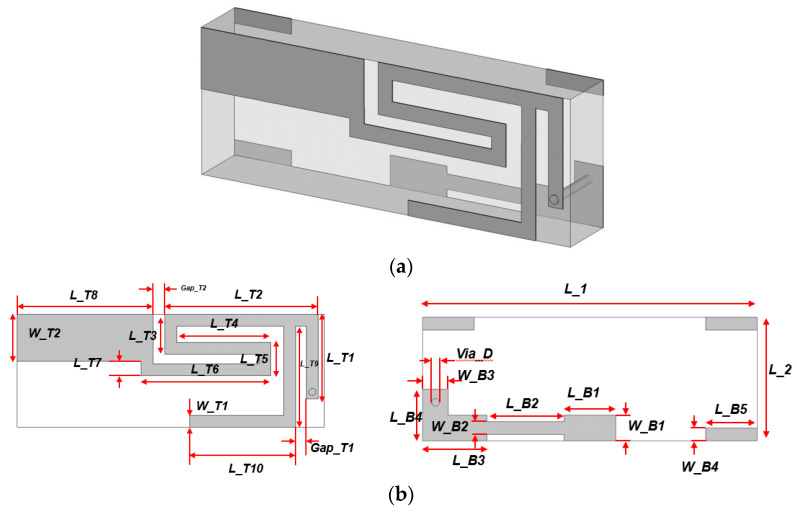

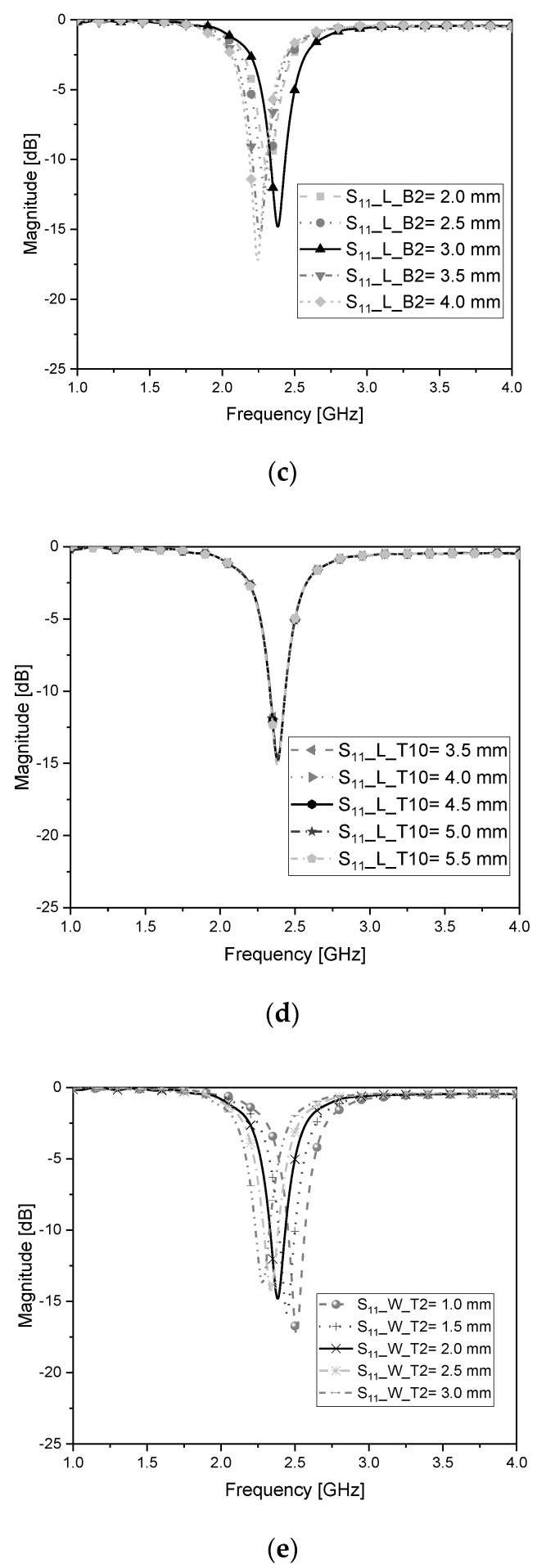

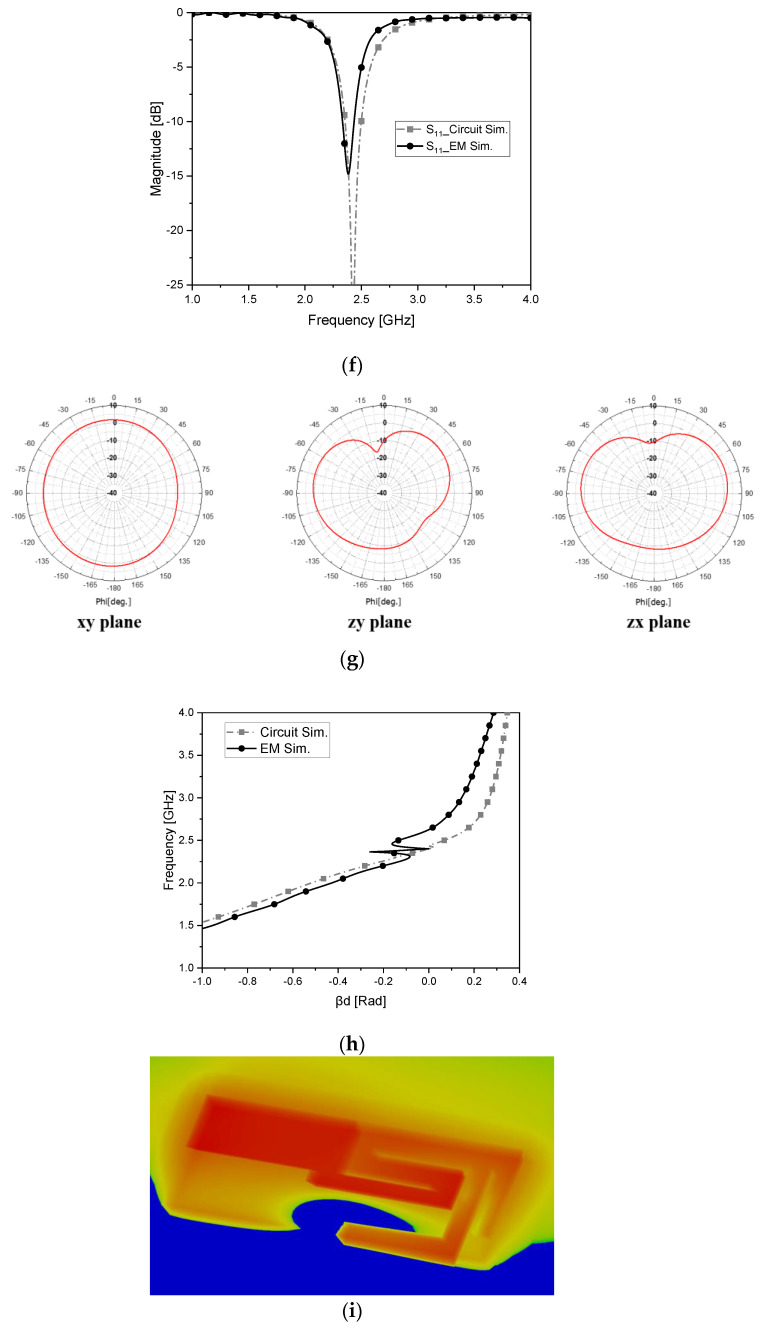
Antenna structure, its EM-simulated frequency-response and metamaterial characteristics. (**a**) Geometrical form in 3D; (**b**) top-view (left) and bottom view (right); (**c**) S_11_ of the antenna; (**d**) S_11_ of the antenna for different L_T10; (**e**) S_11_ of the antenna for different W_T2; (**f**) S_11_ of EM simulation and circuit simulation; (**g**) far-field patterns on xy-, zy- and zx-planes showing the omni-directional effect; (**h**) dispersion diagram of the antenna, (**i**) zeroth-order resonance E-field distribution.

The metalized TX-line segments are formed on the 0.018-mm-thick copper surfaces of FR-4 as a 2-mm thick dielectric material with an ε_r_ of 4.3 and a loss-tangent of 0.025, as in Figure 4a. The structure is given geometrical parameters named *L_T1*, *L_T3*, *L_T5*, *L_T7*, *L_T9*, *W_T1*, *Gap_T1*, *L_T2*, *L_T4*, *L_T6*, *L_T8*, *L_T10*, *W_T2* and *Gap_T2* for the top surface, as seen in the left side of Figure 4b. The top surface is connected through a via to the bottom surface, which will sit on the substrate of the electronic circuit board. The bottom surface, on the right side of Figure 4b, goes with *L_1*, *L_B1*, *L_B3*, *L_B5*, *W_B2*, *Via_D*, *L_2*, *L_B2*, *L_B4*, *W_B1*, *W_B3* and *H*. To comply with the functions of the circuit elements of the E-CRLH model, parametric studies are carried out. As for the main line, for the upper stub and lower stub, that is, the metalized line segments, aiming for resonance at 2.45 GHz, three representative geometrical parameters *L_B2*, *L_T10* and *W_T2* are chosen and adjusted in the course of parametric sweeps. Figure 4c shows the behavior of S_11_ as *L_B2* is varied from 2.0~4.0 mm. Since this parameter is next to the feeding area, this variation increases shunt capacitance and series inductance, which results in the change in the position of the dip. In Figure 4d, though *L_T10* becomes longer, the curve of S_11_ does not move. It is inferred that this parameter makes a loose coupling with the neighboring parameters but helps them with impedance matching. The end part of the main line has *W_T2* as the width and it is varied from 1.0~3.0 mm, as in Figure 4e. Its variation causes a change in resonance frequency. Between the wide and narrow cases, there exists an optimal value for *W_T2* because it works like the element for shunt capacitance and series inductance of the main line, and radiated field as well. After going through this work, the resonance characteristics of the input reflection coefficient from the antenna are obtained, as demonstrated in Figure 4f with the physical dimensions written in the Table 2.

The geometry of the antenna is drawn in CST-MWS as the time-domain full-wave solver discretizing it with hexahedral meshes and an error of |S_11_| being ≤−40 dB to observe, not only S_11_ but the far-field radiated beam as well. It is described by the plots of the beam pattern on the xy-, zy- and zx- planes. Figure 4g demonstrates the polar forms that show a nearly isotropic or omni-directional field distribution, which is expected in the IoT communication. The peak gain is 2.5 dBi. Apart from the frequency response, it is worth seeing the antenna from the metamaterial perspective. As was done in the circuit simulation, the dispersion curve is presented in the EM analysis, as shown in Figure 4h. The LH region as negative *β* is seen in the frequency band lower than the zeroth-order resonance (ZOR or *β* = 0) frequency equal to 2.45 GHz. As another aspect of the metamaterial, the electric field distribution confined to the structure is shown to conform to the ZOR. Figure 4i meets the ZOR effect at 2.45 GHz, like the verification work in reference [27].

## 3. Electromagnetic Tests of the Antenna Alone and Placed in the Earphone

### 3.1. Electromagnetic Connectivity Created and Sensed by the Proposed Antenna

The antenna is fabricated and tested before being incorporated into the PCB in the earphone.

**Figure 5:**
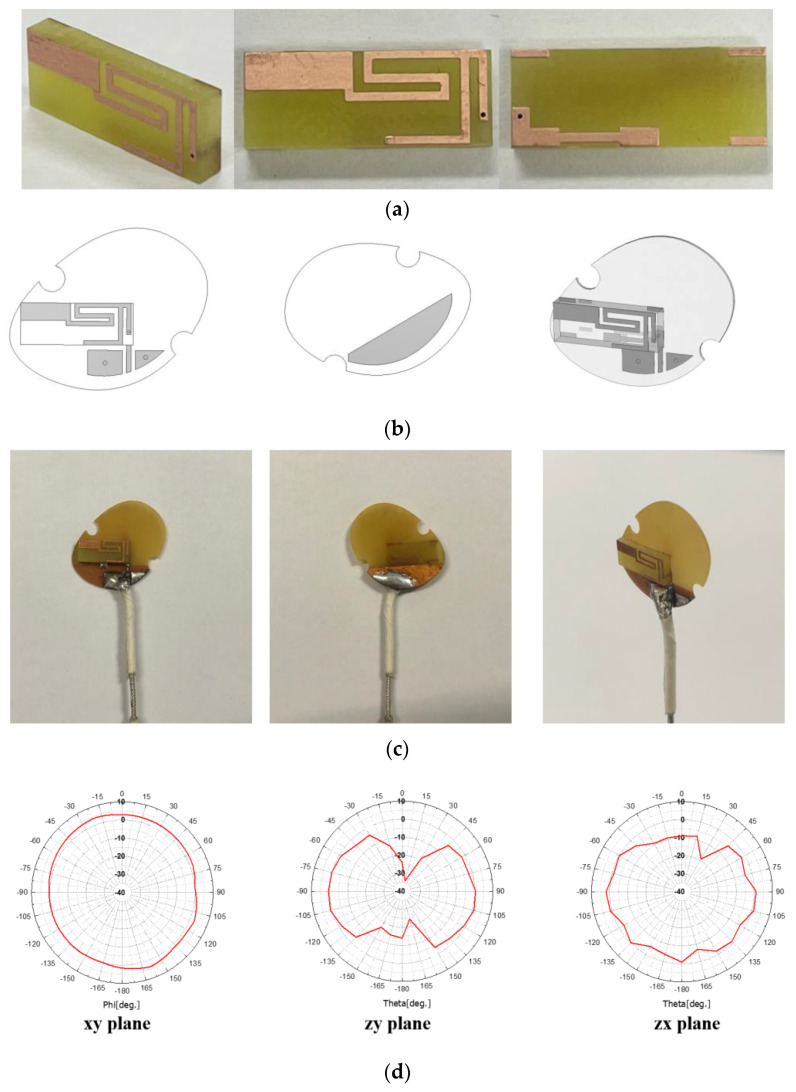
Fabricated antenna and measurement. (**a**) Geometry (**b**) CAD of the antenna on the PCB; (**c**) placing the antenna on the PCB; (**d**) measured far-field patterns on xy-, zy-, zx-planes.

Figure 5a is the manufactured antenna. This is attached to the PCB, as seen in Figure 5b,c. The far-field beam pattern of the antenna is measured, as seen in Figure 5d, which agrees with Figure 4g as the simulated data. Next, this proposed antenna is placed in the earphone mock-up. From this time on, AMCA31-2R450G-S1F-T3, a commercial chip antenna, is compared, regarding the housing and test conditions being the same as the proposed antenna.

**Figure 6:**
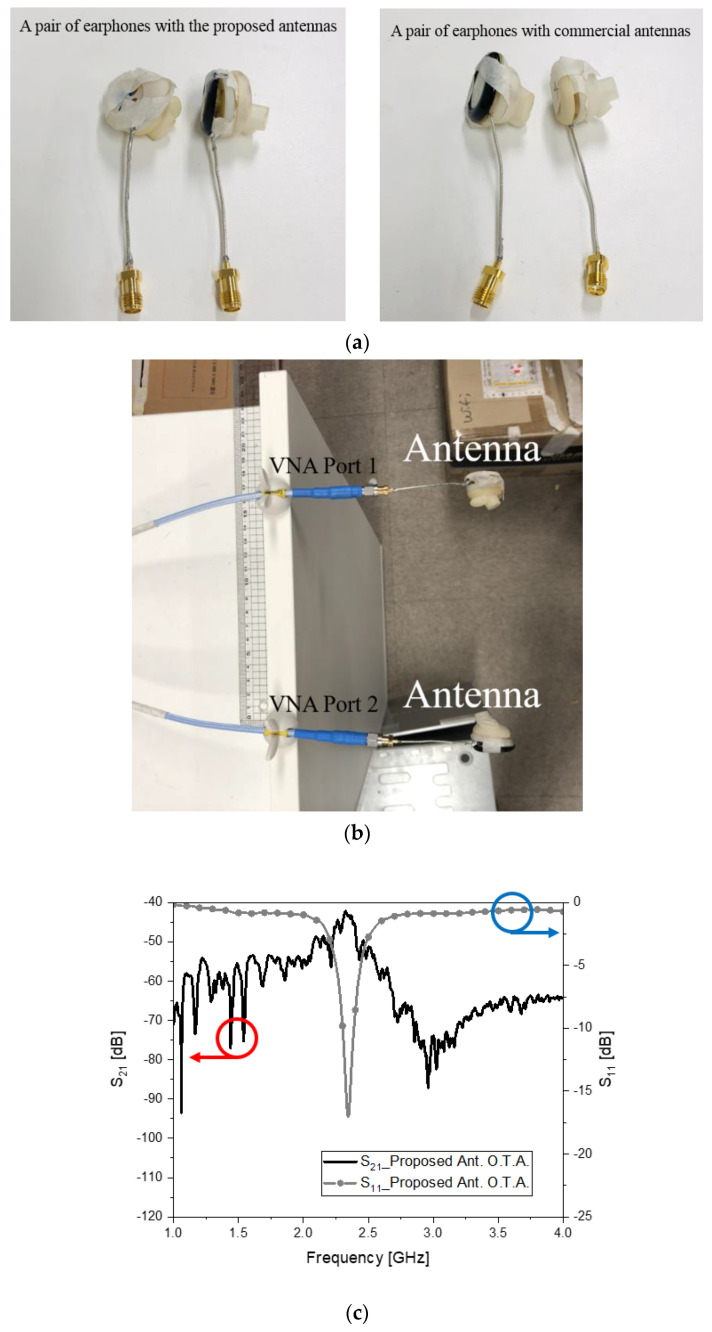

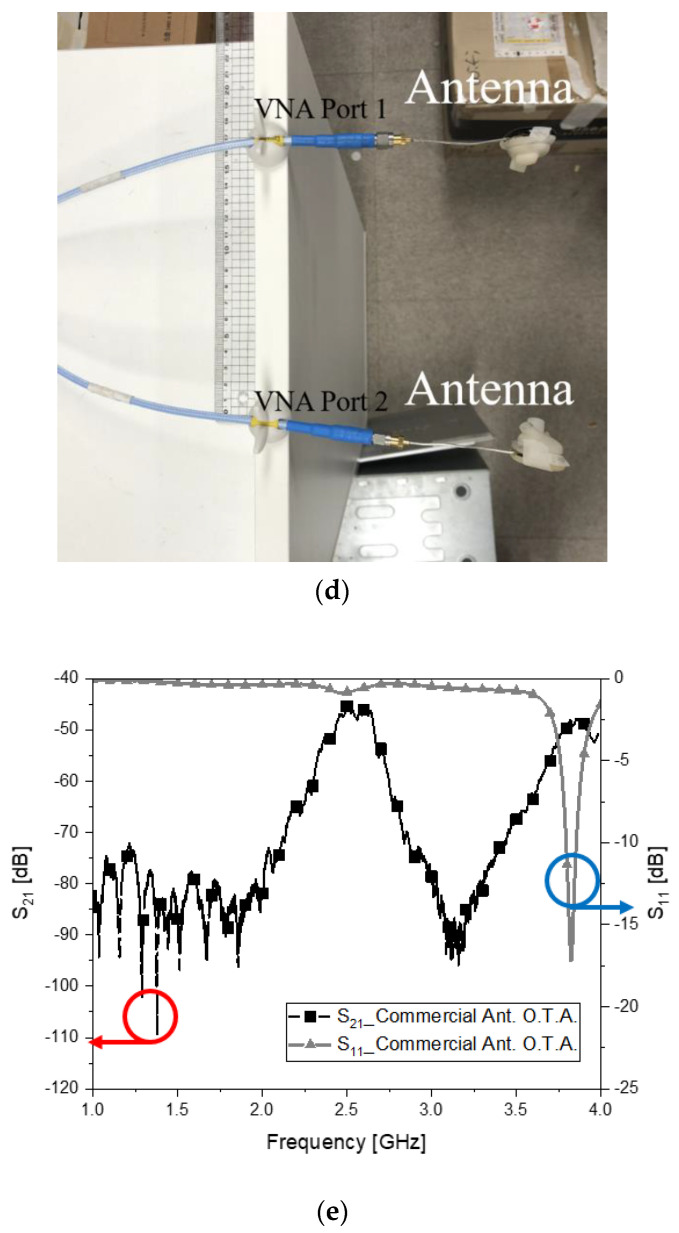
Antennas in the earphones and link test. (**a**) Proposed antennas (left) and commercial antennas (right); (**b**) direct link of the proposed antennas; (**c**) s-parameter of the proposed antennas; (**d**) direct link of the commercial antennas; (**e**) s-parameter of the commercial antennas.

Figure 6a shows the proposed antennas and other antennas, respectively, combined with the earphones. The direct link (over-the-air, OTA) based on resonance leads to S_21_ = −41 dB, which is relatively good connectivity over a 15-cm gap, as seen in Figure 6b,c. Contrarily, the commercial antennas in the earphones have poor resonance results in S_21_ = −45 dB in Figure 6d,e. Therefore, the contributed antenna is superior to the commercial antenna and will be apparent in the next phase of the experiment, which is more realistic.

### 3.2. Electromagnetic Connectivity Test with the Head-Ear-Phantom

The previous test setup is introduced to the head phantom with the ear phantom. POPEYE-V10 is given to this head-ear-phantom and is well known to the bio-EM standardization community. In this test stage, cross-head-interference is examined.

**Figure 7:**
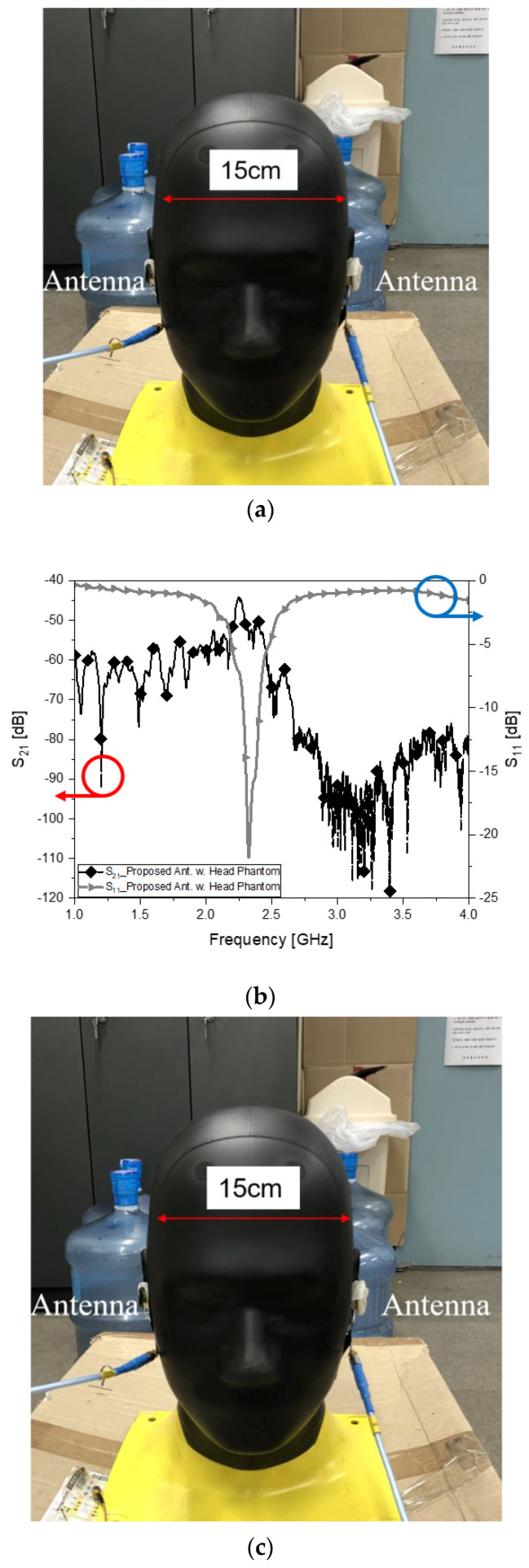

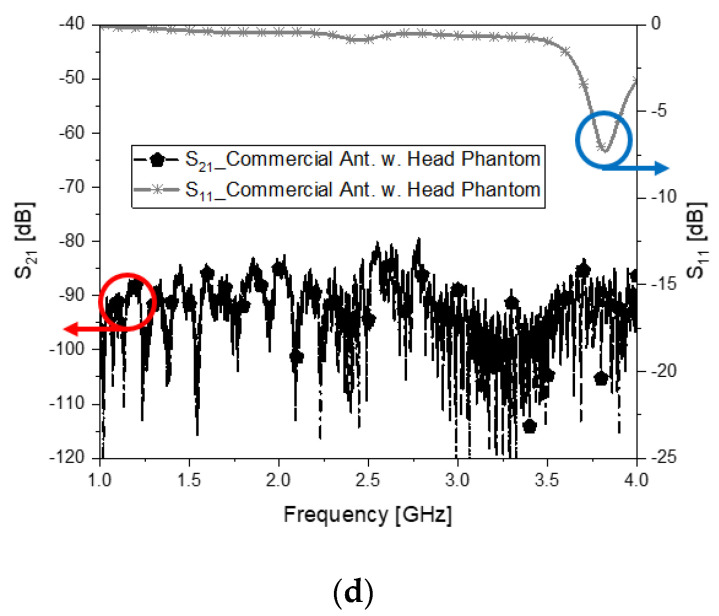
Antennas in the earphones and link test with the head phantom. (**a**) Cross-head link of the proposed antennas; (**b**) s-parameter of the proposed antennas; (**c**) cross-head link of the commercial antennas; (**d**) s-parameter of the commercial antennas.

In this section, an intuitive approach is suggested to get prepared for making antennas robust as well as realizing the effects of human tissue on desirable electromagnetic coupling from one antenna to another. The head phantom wears the earphones with the contributed antennas or commercial antennas. In Figure 7a, the left side of the head is 15 cm apart from the right one; the same as in Section 3.1. The proposed antennas still have good impedance matching as the resonance, and connectivity with S_21_ = −43 dB remains good in Figure 7b, even in the presence of the head SAM. Nevertheless, the commercial antennas embedded in the earphones sitting in the head in Figure 7c present impedance mismatch and a great deal of degradation in wireless connectivity with S_21_ = −85 dB in Figure 7d. In the series of electromagnetic link tests, the proposed antenna part of a wearable device outperforms other antennas represented by the commercial ones. This difference is 40 dB.

## 4. Received Signal Sensitivity Index Test on the Proposed Electromagnetic Sensor

This section displays the first attempt at RSSI measurement with the head and ear phantom for BLT earphones. What is more, is the use of a compact metamaterial antenna. Sensing the electromagnetic fields is translated into the quality of wireless communication by way of RSSI.

**Figure 8:**
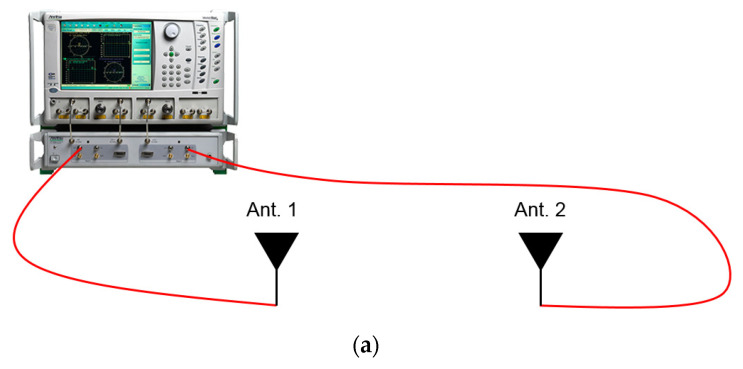

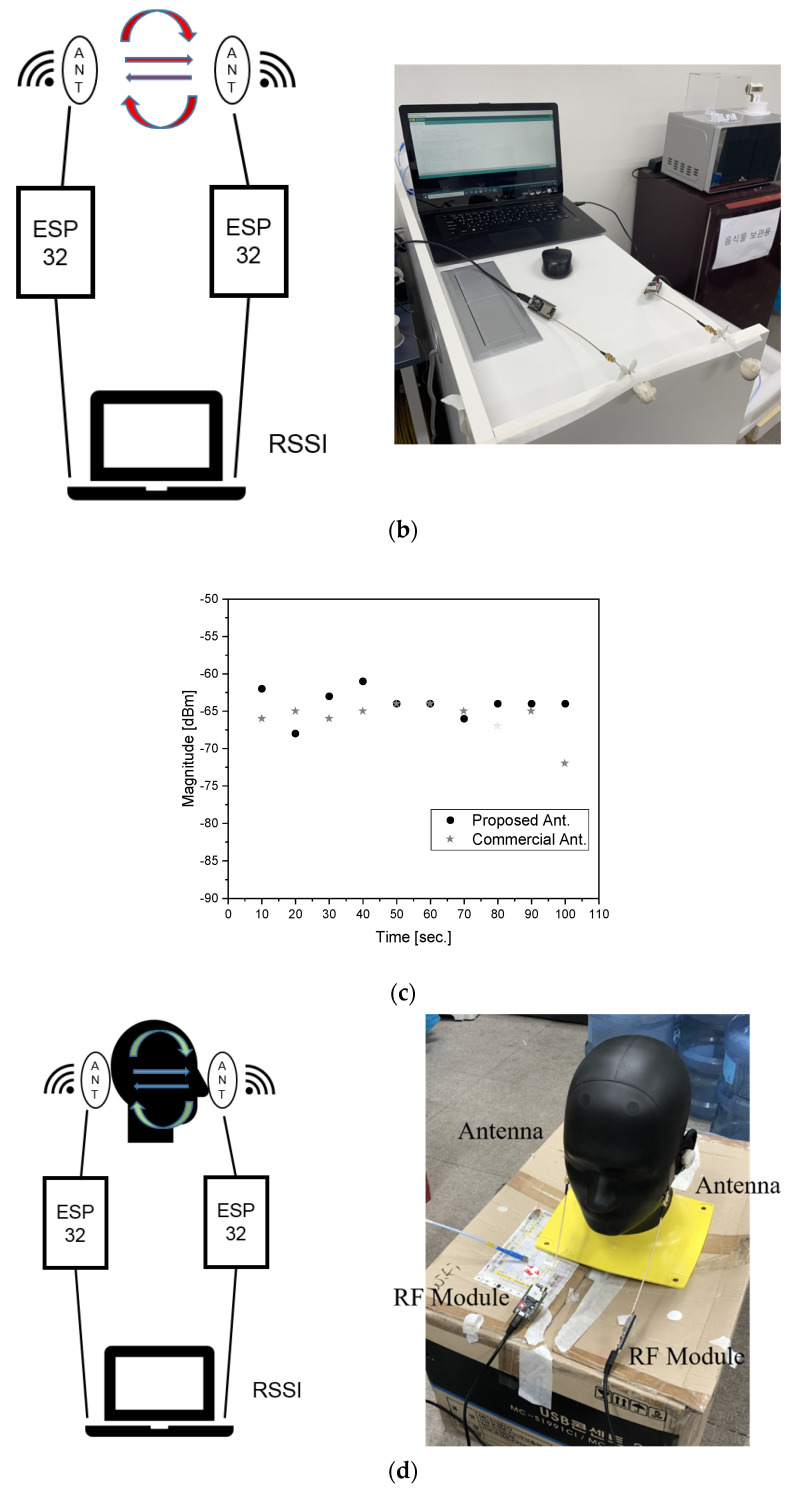

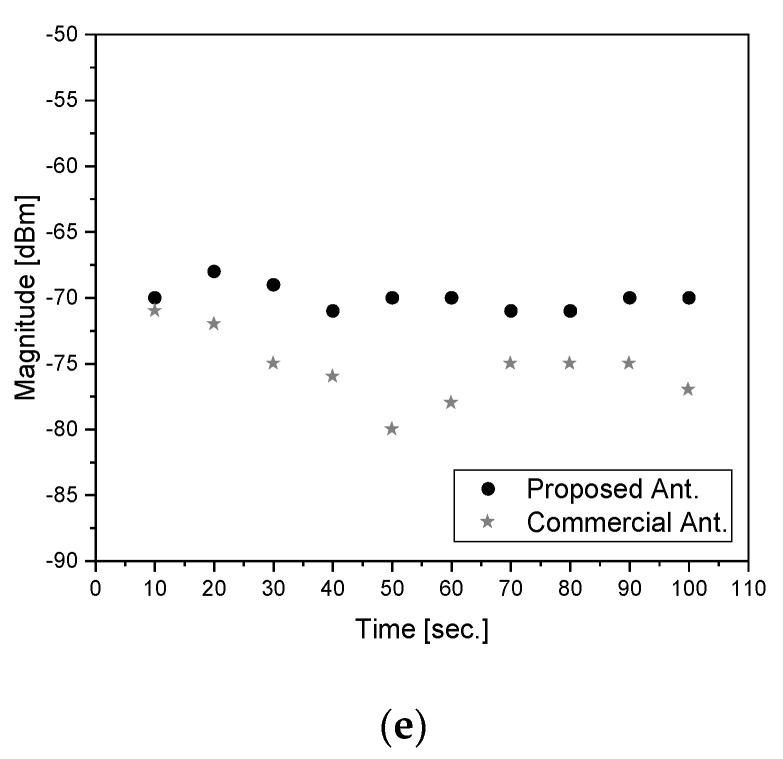
RSSI tests on the earphones without and with the head phantom (**a**) RF-to-RF link test as in Section 3 (**b**) Concept of the RSSI test without the SAM and RSSI (**c**) RSSI of direct link tests (**d**) Concept of the RSSI in the cross-head link and RSSI test (**e**) RSSI of cross-head link tests.

Figure 8a is a scheme of the indirect way to sense wireless link of wearable devices in terms of electromagnetic or RF-to-RF connectivity. This was joined by a vector network analyzer (VNA) displaying S_21_. To evaluate the quality of antenna designs through the system-level view, it is worth measuring the RSSI from the entire chain of communication. Figure 8b depicts the idea and test-bench of RSSI assessment without any obstacle between the earphones. Wi-Fi/Bluetooth modules, named ESP32 chipsets, feed and collect signals for the antennas. In Figure 8c, the RSSI is obtained, ranging from −67.5~−61 dBm with the proposed antennas and −71.2~−63.7 dBm with the commercial ones. Both pairs of antennas work at greater than −120 dBm—the threshold—however, the proposed structure works better. The head-ear-phantom is brought to the test configuration, as demonstrated in Figure 8d. Expectedly, the RSSI falls due to the presence of the SAM. Figure 8e reveals that the proposed antennas are stronger against the cross-head-interference than the commercial ones, whose RSSI falls from −65 dBm to −80 dBm. Furthermore, an additional set of tests are done to investigate the functions of the earphone-mounted antennas without the vector network analyzer, but with batteries. It looks equivalent to the situation where the earphones as wearable devices are powered by batteries, not by long cables.

**Figure 9:**
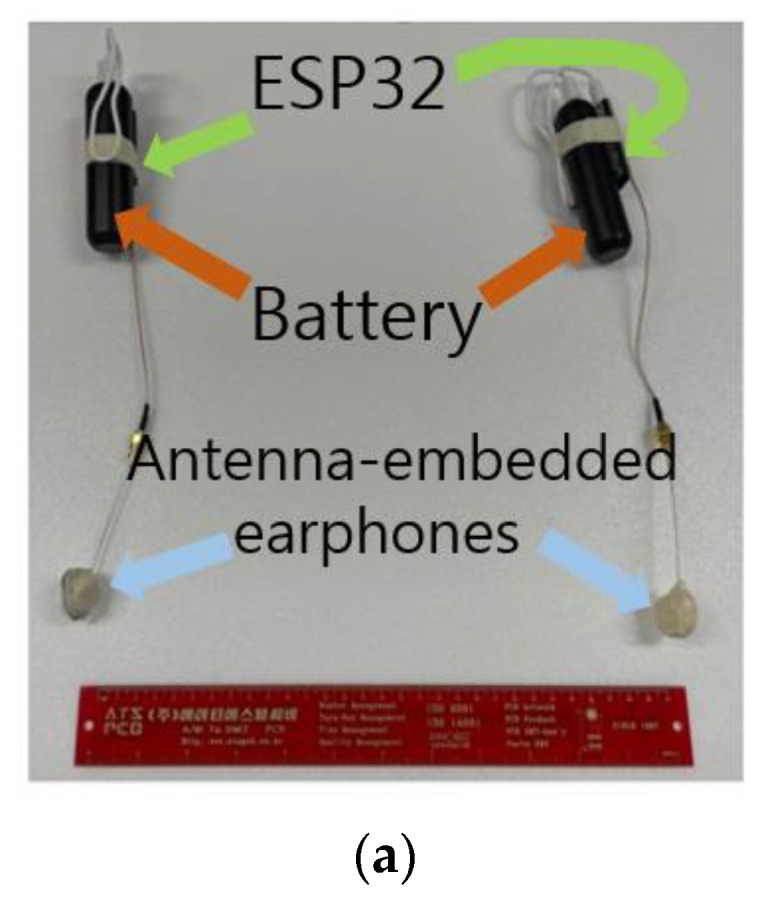

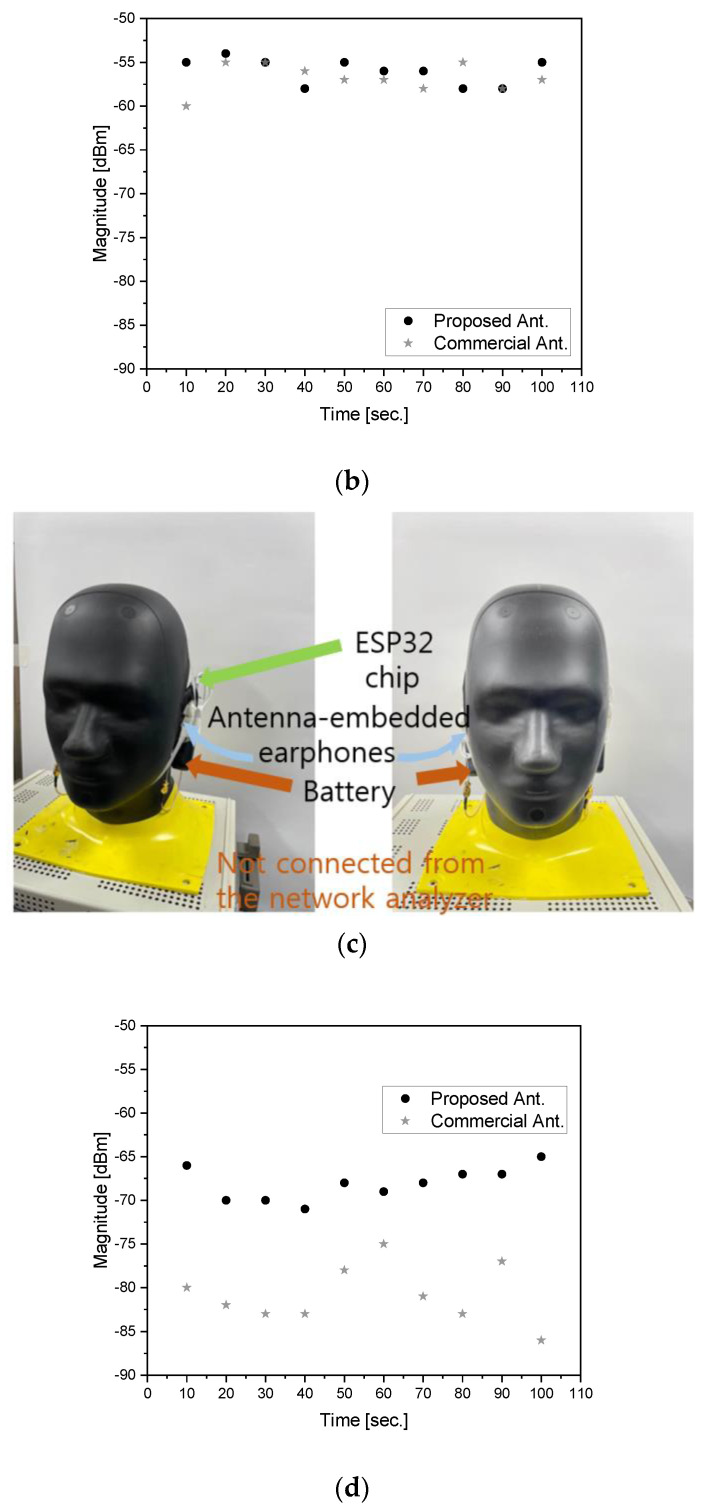
RSSI tests on the earphones powered by batteries. (**a**) RSSI test configuration without the SAM; (**b**) RSSI data; (**c**) RSSI test configuration with the SAM; (**d**) RSSI test results.

As seen in Figure 9a, batteries (5 V and 2 A) are connected to the ESP32 chipsets, which give signals to or take signals from the earphone antennas. Similar to the former tests, the same setup is applied to the proposed antennas and the commercial antennas. Without head-ear-phantom, RSSI curves in the free-space tests are obtained, as seen in Figure 9b, showing the proposed antenna RSSI is higher than the commercial antenna by approximately 2 dB. The RSSI tends to drop when antennas are attached to human tissue. Hence, the two types of earphones powered by the batteries and the BLT chipsets are placed in the ears, as presented in Figure 9c. As expected, the RSSI curves of the two types of antennas become lower than those without the human phantom. It is obvious that the proposed antennas are superior to the commercial antennas by 8 dB to 15.5 dB. It is good to know, therefore, that trying the RSSI test turns out to be useful in assessing IoT antennas performing correctly or incorrectly, and the substantial difference in connectivity can be rooted in the functions of the components.

## 5. Setting a Reference Link and Apparatus Used in the Tests 

In this section, information on the basic characterization method of the antennas as well as the fabrication of the DUTs is given. For the over-the-air and cross-head interference experiments on the BLT earphones, two different types of antennas are employed. One is designed by the authors and the other is a commercial one that is used by the industry of wearable wireless products. The antenna developed by the authors was realized through two steps. Its shape is decided on the basis of the equivalent circuit and its physical dimensions are determined to meet the resonance conditions and the characteristics of the CRLH metamaterial. Additionally, the CAD data are delivered to the PCB manufacturer. The FR-4 glass-reinforced epoxy laminate is chosen as the substrate whose top and bottom surfaces are copper layers. The thickness of the FR-4 and copper is 2 mm and 0.036 mm, respectively. The metalized pattern is formed by removing non-metal areas through the etching process. This antenna is mounted on the base substrate by soldering.

The fabricated antenna and the commercial antenna are brought to a test that they are characterized electromagnetically. In Section 2 and Section 3, their electromagnetic properties were already and sufficiently exhibited, however, another observation to check their wireless features is conducted. The RF transmission coefficient between the metamaterial antenna and a reference device as a popular antenna, entitled National Instruments Corporation’s 2.45 GHz vertical wire antenna, labeled vert2450, is measured to show that the proposed antenna works at the same frequency and delivers an acceptable amount of RF energy at the presence of the reference antenna, defined as the following formula:

(1) $$S_{21}=\frac{P_{Rx:Ref.}}{P_{Tx:DUT}}$$

**Figure 10:**
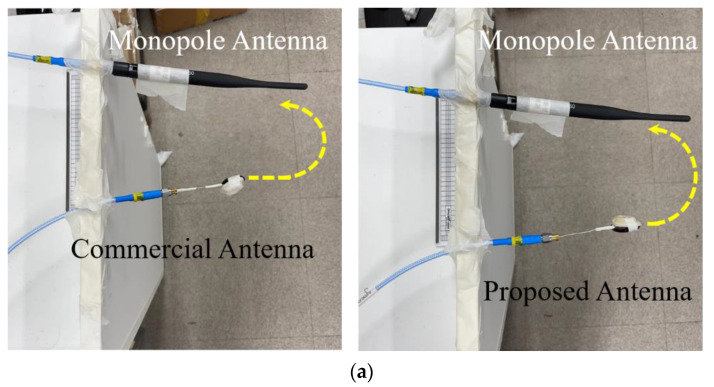

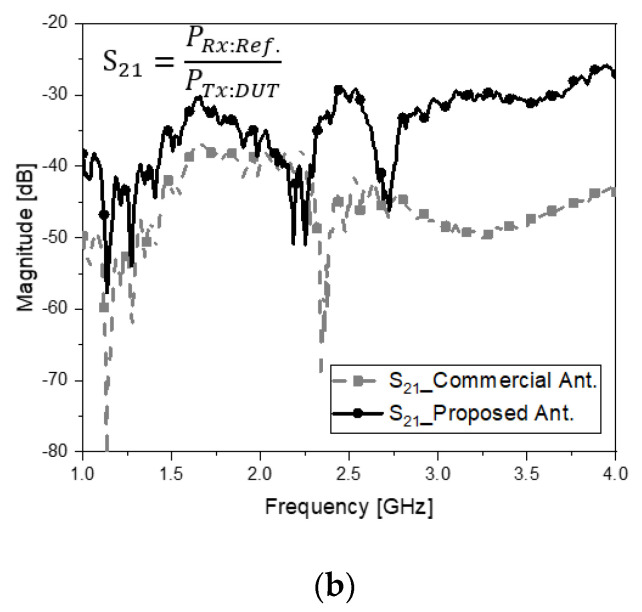
Basic characterization method of the antennas. (**a**) Wire antenna vs. the proposed antenna (left) and wire antenna vs. the commercial antenna (right); (**b**) transmission ratios from the two test cases.

The commercial BLT antenna is characterized in the same manner. The gap between the DUT and the reference antenna is given 10 cm. The left side of Figure 9a presents the proposed antenna and the reference antenna connected to the ports of the VNA through cables, whose model name is 21SK252 3000 mm. On the right side of Figure 10a, the commercial BLT antenna and the reference wire antenna are used for the same type of test. Figure 10b presents that the transmission coefficient of the proposed antenna case is −28 dB which is 17 dB larger than that of the commercial antenna, even though the metamaterial antenna has a smaller size, 13 × 4.8 × 2 mm^3^, than the commercial antenna at 15 × 6 × 1.2 mm^3^.

Aside from the RF technical point of view, the resource of making mock-up covers through the VNA to the human phantom is briefly mentioned.

**Figure 11:**
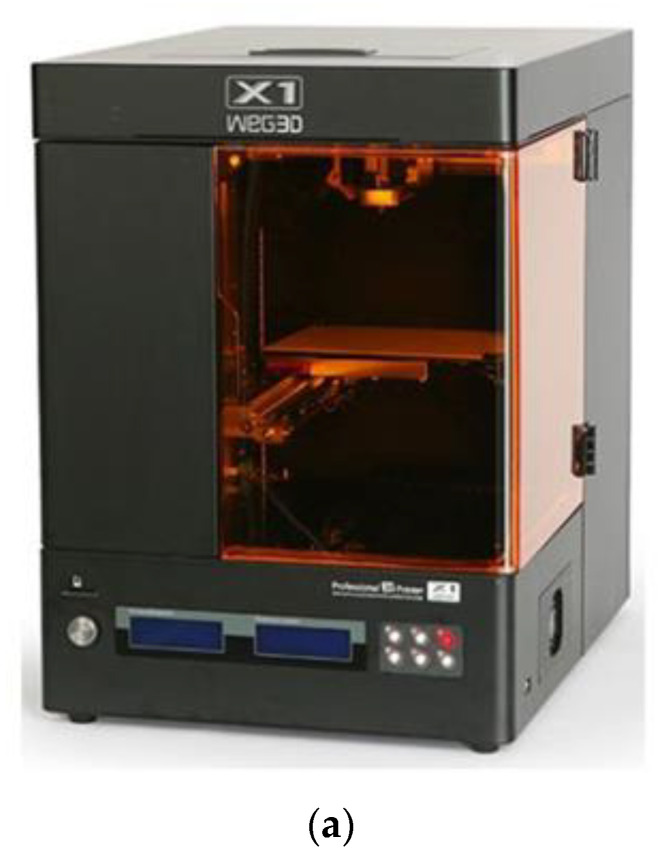

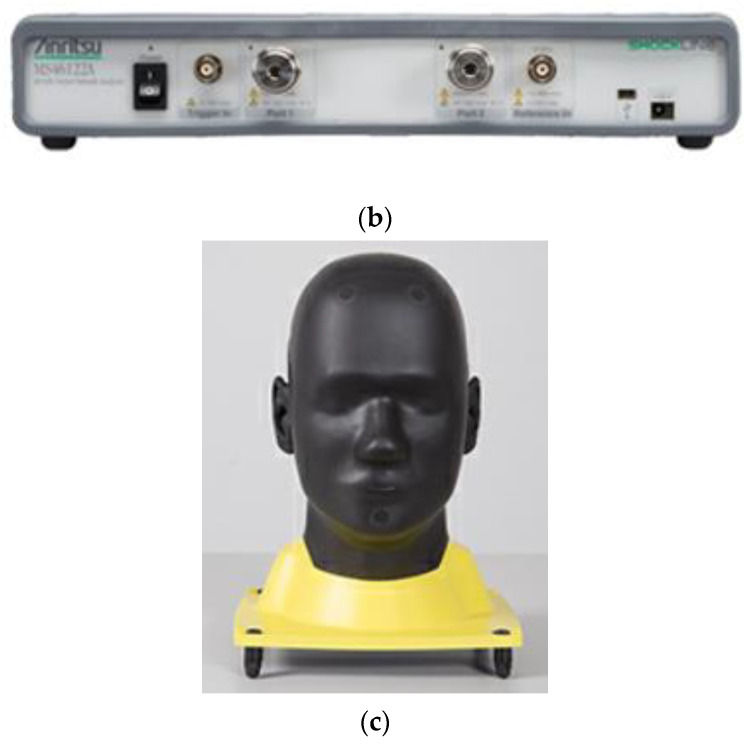
The resource for the tests. (**a**) 3D printer [28]; (**b**) VNA [29]; (**c**) human phantom [30].

The covers of the earphones had to be fabricated by a 3D printer, the WEG3D X1 model of Veltz3D, as shown in Figure 11a. The material of the cover is polylactide (PLA, with a relative permittivity of 2.4). Figure 11b shows that the equipment to measure RF power is the VNA (vector network analyzer), labeled as MS46122A, covering 1 MHz to 43.5 GHz, which was provided by Anritsu Corporation. The head-ear phantom was required to electromagnetically model the human head, as shown in Figure 11c, and its name is HEAD-P10, with a relative permittivity of 25.7 and electric conductivity of 1.32 (S/m) at 2.45 GHz, which was manufactured by Speag.

## 6. Conclusions

A compact antenna is proposed for integration into a Bluetooth earphone, whose shape is in fashion. Based on the E-CRLH metamaterial configuration, this small antenna performs in a proper manner when outside and inside the earphone. Antenna gain is over 1 dBi at 2.45 GHz, with nearly isotropic radiated fields. Furthermore, as a wearable IoT device, when this antenna is loaded onto the ears of the head phantom, S_11_ and S_21_ between the earphones present frequency responses that do not degrade from the free-space case. Furthermore, the performance of the antenna is evaluated from the RSSI test facilitating the BLT wireless chain, including the real chipsets as well as the head-ear-phantom. According to the results, the RSSI is given −67.5~−61 dBm and −70.5~−67.5 dBm, which is compliant with the BLT communication standard, implying that the earphones have the proposed antennas sensing RF signals with little cross-head-interference while the other antennas experience a large scale of degradation. It is predicted that the proposed antenna will carry out its required task in wearable wireless systems.

## Figures and Tables

**Table 1:** The values of the circuit elements of the E-CRLH equivalent model.

Variable Name	Value	Variable Name	Value
*R_s*	50 Ohm	*R_rad*	377 Ohm
*C_se_1*	0.8 pF	*C_sh_1*	0.7 pF
*C_sh_2*	0.6 pF	*C_sh_3*	0.25 pF
*C_sh_4*	1 pF	*L_se_1*	0.8 nH
*L_se_2*	0.5 nH	*L_se_3*	0.3 nH
*L_sh_1*	0.4 nH	*L_sh_2*	0.6 nH

**Table 2:** The values of the geometrical parameters of the antenna. (a) Top surface; (b) bottom surface.

(a)			
Variable Name	Value [mm]	Variable Name	Value [mm]
*L_T1*	3.6	*L_T2*	6.5
*L_T3*	1.7	*L_T4*	4
*L_T5*	1.4	*L_T6*	5.5
*L_T7*	0.6	*L_T8*	5.75
*L_T9*	4.3	*L_T10*	4.5
*W_T1*	0.5	*W_T2*	2
*Gap_T1*	0.5	*Gap_T2*	0.45
**(b)**			
**Variable Name**	**Value [mm]**	**Variable Name**	**Value [mm]**
*L_1*	13	*L_2*	4.8
*L_B1*	2	*L_B2*	3
*L_B3*	2.5	*L_B4*	2
*L_B5*	2	*W_B1*	1
*W_B2*	0.5	*W_B3*	1
*Via_D*	0.3	*H*	2

## Data Availability

Not applicable.
